# Genomic insight into pathogenicity of dematiaceous fungus *Corynespora cassiicola*

**DOI:** 10.7717/peerj.2841

**Published:** 2017-01-26

**Authors:** Hong Keat Looi, Yue Fen Toh, Su Mei Yew, Shiang Ling Na, Yung-Chie Tan, Pei-Sin Chong, Jia-Shiun Khoo, Wai-Yan Yee, Kee Peng Ng, Chee Sian Kuan

**Affiliations:** 1 Department of Medical Microbiology, University of Malaya, Kuala Lumpur, Malaysia; 2 Department of Science and Technology, Codon Genomics SB, Seri Kembangan, Selangor, Malaysia

**Keywords:** *Corynespora cassiicola*, Pathogenicity, CAZyme, Appressoria, Toxin, Virulence factor, Melanin

## Abstract

*Corynespora cassiicola* is a common plant pathogen that causes leaf spot disease in a broad range of crop, and it heavily affect rubber trees in Malaysia (Hsueh, 2011; Nghia et al., 2008). The isolation of UM 591 from a patient’s contact lens indicates the pathogenic potential of this dematiaceous fungus in human. However, the underlying factors that contribute to the opportunistic cross-infection have not been fully studied. We employed genome sequencing and gene homology annotations in attempt to identify these factors in UM 591 using data obtained from publicly available bioinformatics databases. The assembly size of UM 591 genome is 41.8 Mbp, and a total of 13,531 (≥99 bp) genes have been predicted. UM 591 is enriched with genes that encode for glycoside hydrolases, carbohydrate esterases, auxiliary activity enzymes and cell wall degrading enzymes. Virulent genes comprising of CAZymes, peptidases, and hypervirulence-associated cutinases were found to be present in the fungal genome. Comparative analysis result shows that UM 591 possesses higher number of carbohydrate esterases family 10 (CE10) CAZymes compared to other species of fungi in this study, and these enzymes hydrolyses wide range of carbohydrate and non-carbohydrate substrates. Putative melanin, siderophore, *ent*-kaurene, and lycopene biosynthesis gene clusters are predicted, and these gene clusters denote that UM 591 are capable of protecting itself from the UV and chemical stresses, allowing it to adapt to different environment. Putative sterigmatocystin, HC-toxin, cercosporin, and gliotoxin biosynthesis gene cluster are predicted. This finding have highlighted the necrotrophic and invasive nature of UM 591.

## Introduction

*Corynespora cassiicola* is an ascomycete which is well-known for its pathogenicity to a wide range of plants in tropical and subtropical countries (Dixon et al., 2009). This fungus is notorious for causing spot diseases in economically important crop (such as cowpea, cucumber, papaya, rubber, soybean, and tomato) (Dixon et al., 2009), leaf fall disease in rubber trees (Nghia et al., 2008), and petal spots in Hydrangea (Leite & Barreto, 2000). In year 2000, spores of *C. cassiicola* has been identified as part of the airborne allergens that triggers allergenic responses in patients having respiratory problem (Yi et al., 2000). In rare occasions, this phytopathogen infects human and causes keratitis (Yamada et al., 2013), maduromycetoma (Mahgoub, 1969), subcutaneous infection (Huang et al., 2010), and phaeohyphomycosis (Lv et al., 2011; Wang et al., 2014).

Over the recent four decades, the number of reported *C. cassiicola* infections in human are limited to less than ten cases, but this opportunistic fungus still causes extensive skin damage upon successful infection. Majority of the patients that contracted the fungus are involved in agriculture work, regardless of whether the host is immunity-compromised (Huang et al., 2010; Yamada et al., 2013), wounded (Lv et al., 2011), or physically healthy and immunocompetent (Yamada et al., 2013). In this study, we identified a strain of *C. cassiicola*, namely *C. cassiicola* UM 591, that was isolated from a patient’s contact lens, whom was diagnosed with ocular mycosis.

The first obstacle in fungal invasion is the host’s natural defence system. In the plant, the cell wall is the best defence against fungal invasion (Smart, 1991). In warm-blooded organisms, endothermy has been suggested as an additional layer of natural defence from fungal invasion aside from skin barrier and immune system (Casadevall, 2007). In order to overcome these obstacles, fungi adopt variable means of invasion, which can be grouped into three main categories; enzymatic, chemical, and physical effectors.

Enzymatic effectors include plant degradative enzymes, secreted peptidases, and lipases. Plant pathogens are equipped with diverse classes of Carbohydrate-Active enZymes (CAZymes) (Cantarel et al., 2009), which include cellulases, hemicellulases, pectinases, and cutinases (Zhao et al., 2014). These enzymes facilitate fungal invasion via degradation or modification of plant’s cell wall. On the other hand, human pathogens require lipases and peptidases to degrade keratinized medium (such as hair, skin or nail) and cell’s lipid bilayer (Hogan, Klein & Levitz, 1996). Cysteine peptidases and bacterial-derived phospholipases have been found capable of interfering host’s immune system by inhibition or degradation of immune response pathway components in the former (Donnelly, Dalton & Robinson, 2011; Kobayashi et al., 1993) and disruption of cell signalling pathway in the latter (Bender & Flieger, 2010).

Fungus produces a broad range of secondary metabolites (Howlett, 2006), peptides or chemicals to survive in nutrient-deficit or constrained environment (Al-Fakih, 2014). Melanin is a common secondary metabolite biosynthesized by dematiaceous fungi to protect themselves from UV and chemical stresses (Revankar & Sutton, 2010) as well as aiding in appressorial-dependent host infection (Pihet et al., 2009). Toxins are the hallmark of necrotrophic fungi and play important roles in procuring nutrient and colonization (Howlett, 2006). On the other hand, fungal terpenoids could be either toxic (Collado, Sánchez & Hanson, 2007) or serve a defensive purpose (Avalos & Limón, 2015).

Aside from the leaves and fruits, *C. cassiicola* also infects root, hypocotyl, boll, and stem; leading to rot diseases (Ahmed, Alam & Khair, 2014; Lakshmanan, Jeyarajan & Vidhyasekaran, 1990; Seaman, Shoemaker & Peterson, 1965). *C. cassiicola* is a host-selective phytopathogen, but it has been observed that most of the *C. cassiicola* strains are highly pathogenic to tomatoes (Onesirosan et al., 1975; Qi et al., 2011). This fungus grows optimally on potato dextrous agar (PDA) and soybean root at 25 °C and 15 °C, respectively (Ahmed, Alam & Khair, 2014; Seaman, Shoemaker & Peterson, 1965). Seaman, Shoemaker & Peterson (1965) also reported that *C. cassiicola* loses its pathogenicity on soybean root when soil temperature rises above 20 °C, denoting that this fungus requires optimal condition and environment to exhibit its pathogenicity.

In the few reported cases of mycosis, necrotic lesions were observed (Huang et al., 2010; Lv et al., 2011), and *C. cassiicola* had been considered to be potentially angioinvasive (Lv et al., 2011). So far, treatments have been successful with terbinafine (MIC: 0.125 µg/mL), 5% povidone iodine, amphotericin B (MIC: 0.5 µg/mL), and voriconazole (MIC: 0.015 µg/mL) (Huang et al., 2010; Lv et al., 2011; Yamada et al., 2013). Cycloheximide and 5% povidone iodine (at 1:256 dilution) have been reported to inhibit fungal growth (Lv et al., 2011), and other potentially potent antifungal drugs against *C. cassiicola* includes butenafine (MIC: 0.25 µg/mL) and micafungin (MIC: ≤0.03 µg/mL) (Lv et al., 2011; Yamada et al., 2013).

*C. cassiicola* has been studied a lot in plant research, but there are little information that describe the underlying factors that contribute to its pathogenicity. In addition, there are a handful of reported cases of human infection caused by this fungus, but very little publications that describe its pathogenicity in human. In light of the availability of a clinical strain, we are interested in deciphering the potential virulence factors in *C. cassiicola* that enable this fungus to be necrotic in both plant and human. We speculate that there may be similarity in gene expression among dermatophytes that could be potential virulence factors that we can address via a predictive study. We carried out a genome sequencing on UM 591, and subsequently, these bioinformatics data are subjected to homology searches in publicly available bioinformatics databases to annotate and predict putative virulence genes or proteins for more detailed description and analyses. The findings in this study will provide better understanding on *C. cassiicola*, its virulence factors, and other genetic variances that helps this fungus to survive and proliferate on human skin, which is not a common habitat or host for this fungus.

## Materials and Method

### Ethics statement

The fungal isolate, UM 591, was obtained from the archived fungal collection in University Malaya Medical Centre (UMMC). The clinical isolate was made anonymous, and the only information that was retained were the source of isolation and clinical diagnosis. No demographic, clinical history, or patient’s information was retained. Therefore, no traceable data pertaining to patient’s identity is available and the study is exempted from ethical approval by our teaching hospital (http://umresearch.um.edu.my/doc/File/UMREC/6_CODE%20OF%20RESEARCH%20ETHICS%20%20IN%20UNIVERSITY%20OF%20MALAYA.pdf).

### Fungal isolate

UM 591 was isolated from the contact lens of a patient diagnosed with keratomycosis in UMMC, Kuala Lumpur, Malaysia. The isolate was processed as described previously (Yew et al., 2014) and was cultured on Sabouraud Dextrose Agar (SDA) up to 7 days at 30 °C with regular observation on alternate days for growth progress.

### ITS sequencing and phylogenetic analysis

The internal transcriber spacer (ITS) region was used for molecular taxonomy identification of UM 591. Briefly, total DNA extraction, PCR amplification, and DNA sequencing were carried out as described previously (Yew et al., 2014). ITS1 (5^′^-TCC GTA GGT GAA CCT GCG G-3^′^) and ITS4 (5^′^-TCC TCC GCT GCT TAT TGA TAT GC-3^′^) primer pair were used for PCR amplification with annealing temperature of 58 °C. The sequencing data was subjected to BLASTn homology search against the UNITE nucleotide database (Kõljalg et al., 2013) for curated fungal species identification. Unique ITS nucleotide sequence from the isolate, reference *Corynespora* species, and an outgroup strain of *Falciformispora lignatilis* (Table S1) were subjected to phylogenetic analysis. In brief, the phylogenetic analysis was performed using MrBayes based on Bayesian Markov Chain Monte Carlo (MCMC) algorithm. The analysis was conducted by sampling across the entire general time reversible (GTR) model space (Ronquist & Huelsenbeck, 2003). ITS alignments were carried out with sampling frequency of 100 for a total of 500,000 generations, and diagnostics were calculated for every 1,000 generations. A burn-in setting of 25% was used to discard the first 1,250 trees. A standard deviation of split frequencies below 0.01 was used to assess convergence. The plot of generation versus log probability of the data did not show noticeable trend, and potential scale reduction factor (PSRF) close to 1.0 was set for all parameter.

### Genomic DNA extraction, genome sequencing, and *de novo* assembly

Genomic DNA of UM 591 was extracted as described previously (Kuan et al., 2015) and the fungal genome sequencing was carried out using Illumina HiSeq 2000 Sequencer (Illumina Inc. San Diego, CA, USA) in a 2 × 90 bp paired-end mode with library insert sizes of 500 bp (short insert, SI) and 5-kb (long insert, LI). All sequence reads were pre-processed using FASTX-Toolkit (http://hannonlab.cshl.edu/fastx_toolkit/). Two (for short insert) and three (for long insert) bases were removed from 5^′^-terminal of the reads. Bases from 3^′^-end of the reads that have Phred quality below Qv20 were trimmed and reads less than 50 bp were removed. Reads having ≥40% bases with Qv ≤ 20 were removed as well. Pre-processed reads from both libraries were *de novo* assembled by Velvet v1.2.08 with the following parameters: k-mer setting = 75, insert length = 458, -ins_length_sd = 100, insert_length2 = 5,472, ins_ length2_sd = 1,400. Additional parameter was set for large insert library as follows: -shortMatePaired = yes. Scaffolds were built from contigs assembled with Velvet using SSPACE Basic v2.0 and following parameters: −*z* 100, −*k* 15, −*a* 0.3 and −*n* 30. Gap filling was performed using GapFiller v1.10 with paired-end sequencing data from both libraries and the following parameters: −*m* = 60, −*o* = 15, −*r* = 0.8, −*n* = 30, −*t* = 30 and −*T* =10). The assembled genome was then assessed for completeness using BUSCO v1.22 with BUSCO profiles for fungi (Simão et al., 2015).

### Gene prediction and annotation

Interspersed repetitive elements and low complexity DNA sequences of UM 591 were masked using RepeatMasker version open-4.0.5 with RepeatMasker Database release 20140131 (http://www.repeatmasker.org), followed by rRNA and tRNA detection using RNAmmer v1.2 (Lagesen et al., 2007) and tRNAscan-SE v1.3.1 (Lowe & Eddy, 1997), respectively. The rRNAs and tRNAs identified were then masked from the genome using maskFastaFromBed of Bedtools v2.16.2 (Quinlan & Hall, 2010). Prediction of protein coding gene were carried out on repeat- and RNA-masked genome using GeneMark-ES v2.3e with branch point setting (–BP) turned on by default (Ter-Hovhannisyan et al., 2008). The completeness of gene prediction was assessed using BUSCO v1.22 with fungi profiles (Simão et al., 2015). The predicted coding sequences with ≥99 nt were searched against NCBI (nt) and Swiss-Prot protein databases by using BLASTX v2.2.28+, with e-value ≤ 1e–05. The top 20 NR blast hits and top 20 Swiss-Prot blast hits were then analyzed using Blast2GO v2.6.3 (Conesa et al., 2005) for GO term annotation and KEGG pathway mapping. Functional classification of the predicted proteins (≥33 aa) was performed using KOG (Tatusov et al., 2003) while motifs and protein domains were determined with InterProScan v5 (Jones et al., 2014), searches against InterPro databases, including Pfam, PRINTS, PROSITE, PANTHER and SMART.

Predicted protein models of UM 591 and 16 fungal species (Table S1) were submitted to databases of automated Carbohydrate-active enzyme ANnotation (dbCAN) (Yin et al., 2012) for the identification of Carbohydrate-Active enZyme (CAZyme). A comparative analysis was carried with these CAZyme data. All the fungal genomes were selected based on nutritional strategy each species adopt as described previously (Zhao et al., 2014), with the exception of mycosis-causing species (*Trichophyton rubrum* CBS118892, *Microsporum gypseum* CBS118893, and *Candida albicans* SC5314) and our laboratory isolate (UM 591, *Bipolaris papendorfii* UM 226, *Ochroconis mirabilis* UM 578, and *Daldinia eschscholtzii* UM 1020). Peptidases were identified using MEROPS database (Rawlings, Barrett & Bateman, 2012). Secreted peptides and transmembrane domains were predicted using SignalIP version 4.1 (Petersen et al., 2011) and TMHMM version 2.0 (Krogh et al., 2001), respectively. Putative secreted peptides were identified based on the presence of signal peptide secretion signal and absence of transmembrane domain (except for 40 amino acids at N-terminal, which mark for peptide secretion sequence). Lipases were predicted by amino acids sequence search in Lipase Engineering database (Ohm et al., 2012). Secondary metabolite gene clusters were predicted using Secondary Metabolite Unique Regions Finder (SMURF: http://www.jcvi.org/smurf/index.php) and secondary metabolite domains were annotated using InterProScan. Genes homologous to known virulent factor genes were identified using BLAST search with the protein model in pathogen-host interaction database (PHI-base) (http://www-phi4.phibase.org/).

## Result and Discussion

### Identification of UM 591 isolate

**Figure 1:**
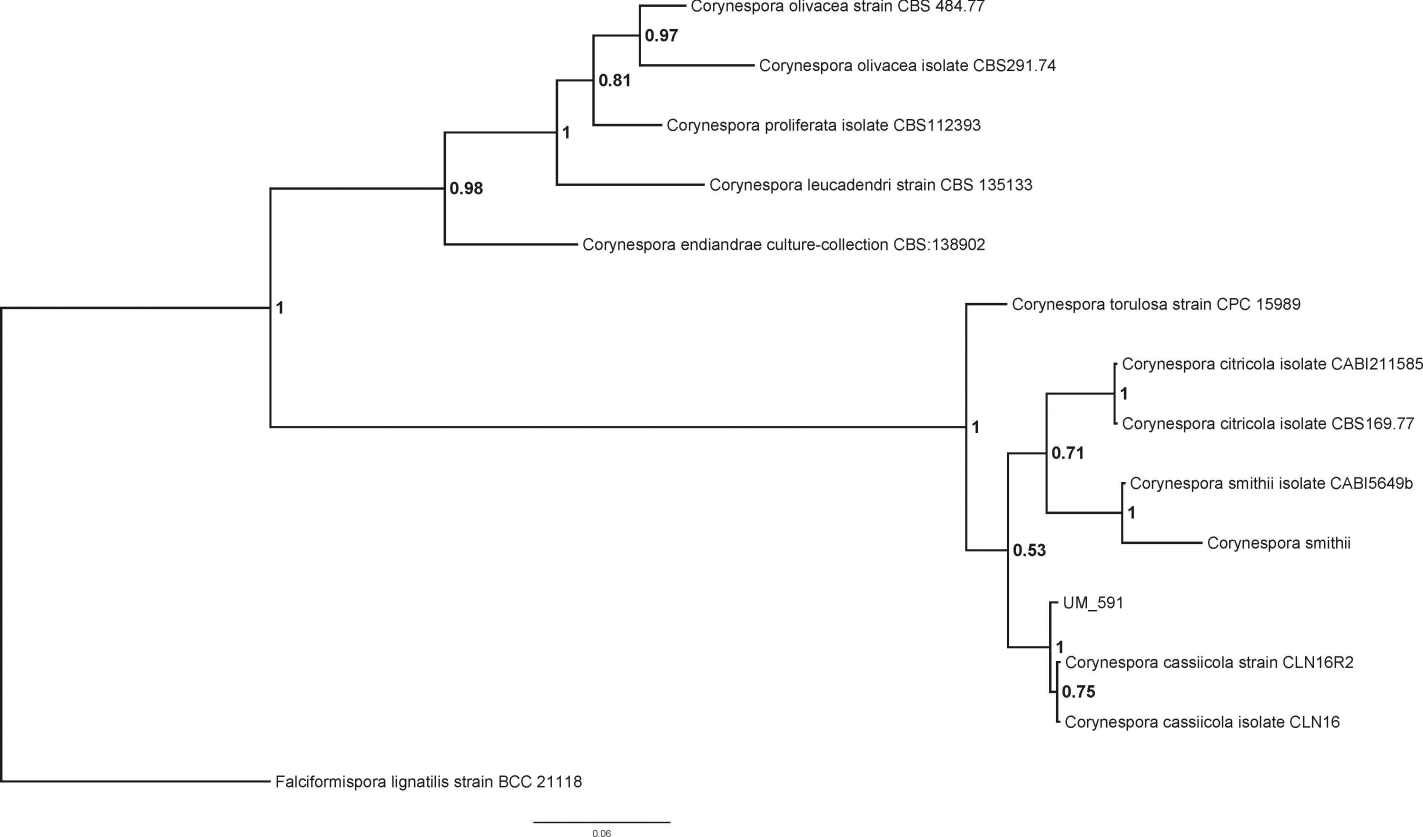
Bayesian phylogram generated using ITS sequence of 13 reference fungus as mentioned previously. The tree is rooted with *Falciformispora lignatilis* strain BCC21118 as outgroup. The number on the nodes indicate Bayesian posterior probability based on sampling frequency of 100 for a total of 500,000 generations.

The identity of UM 591 isolate could not be determined based on its colonial and microscopy morphology (data not shown). The preliminary morphological identification of the UM 591 isolate was confirmed by PCR amplification of the ITS gene region and ITS-based phylogeny. Homology search of UM 591 *ITS1* sequence in UNITE database return a total of 15 alignments that matches *Corynespora cassiicola*. The top three alignments belong to *Corynespora cassiicola* strain 6M (Genbank: JX087444, 99% identical, bit score: 1063), *Corynespora cassiicola* isolate YP59 (Genbank: FJ852716, 100% identical, bit score: 1007), and *Corynespora cassiicola* isolate YP42 (Genbank: FJ852714, 100% identical, bit score: 1007). *ITS1* sequence of *C. citricola* CBS169.77, *C. citricola* CABI211585, *C. endiandrae* CBS138902, *C. leucadendri* CBS135133, *C. olivacea* CBS484.77, *C. olivacea* CBS291.74, *C. proliferata* CBS112393, *C. smithii*, *C. smithii* CABI5649b, *C. torulosa* CPC15989, *C. cassiicola* CLN16, and *C. cassiicola* CLN16R2 were used to construct a phylogram with *Falciformispora lignatilis* strain BCC21118 as an out-group (Fig. 1). The result from the phylogenetic analysis shows that UM 591 falls into the cluster of *C. cassiicola* species, within the same clade as *C. smithii* and *C. citricola* (Fig. 1). The phylogenetic analysis has confirmed that the UM 591 belongs to the species *C. cassiicola*.

### Genome feature of *Corynespora cassiicola* UM 591

Genome sequencing of UM 591 has generated a total of 1,962 contigs with an assembly size of 41.8 Mb. The draft genome consists of 1,941 contigs (≥200 bp) with average GC content of 51%. These contigs are assembled into 189 scaffolds (≥1,000 bp) with N50 value of 1,460 kb. Genome statistics of UM 591 is shown in Table 1.

**Table 1:** Genome statistics of *Corynespora cassiicola* UM 591.

*Corynespora cassiicola* UM 591	Statistical data
Total number of reads of 500 bp insert	33,600,000
Total number of reads of 5-kb insert	22,277,778
Read length (bp)	90
Assembly size (Mb)	41.88
Contigs (≥200 bp)	1,941
Contigs (N50) (kb)	44,124
Number of scaffolds (≥1,000 bp)	189
Scaffold (N50) (kb)	1,460
Contigs G + C (%)	51.86

### Genome and gene functional annotation

**Figure 2:**
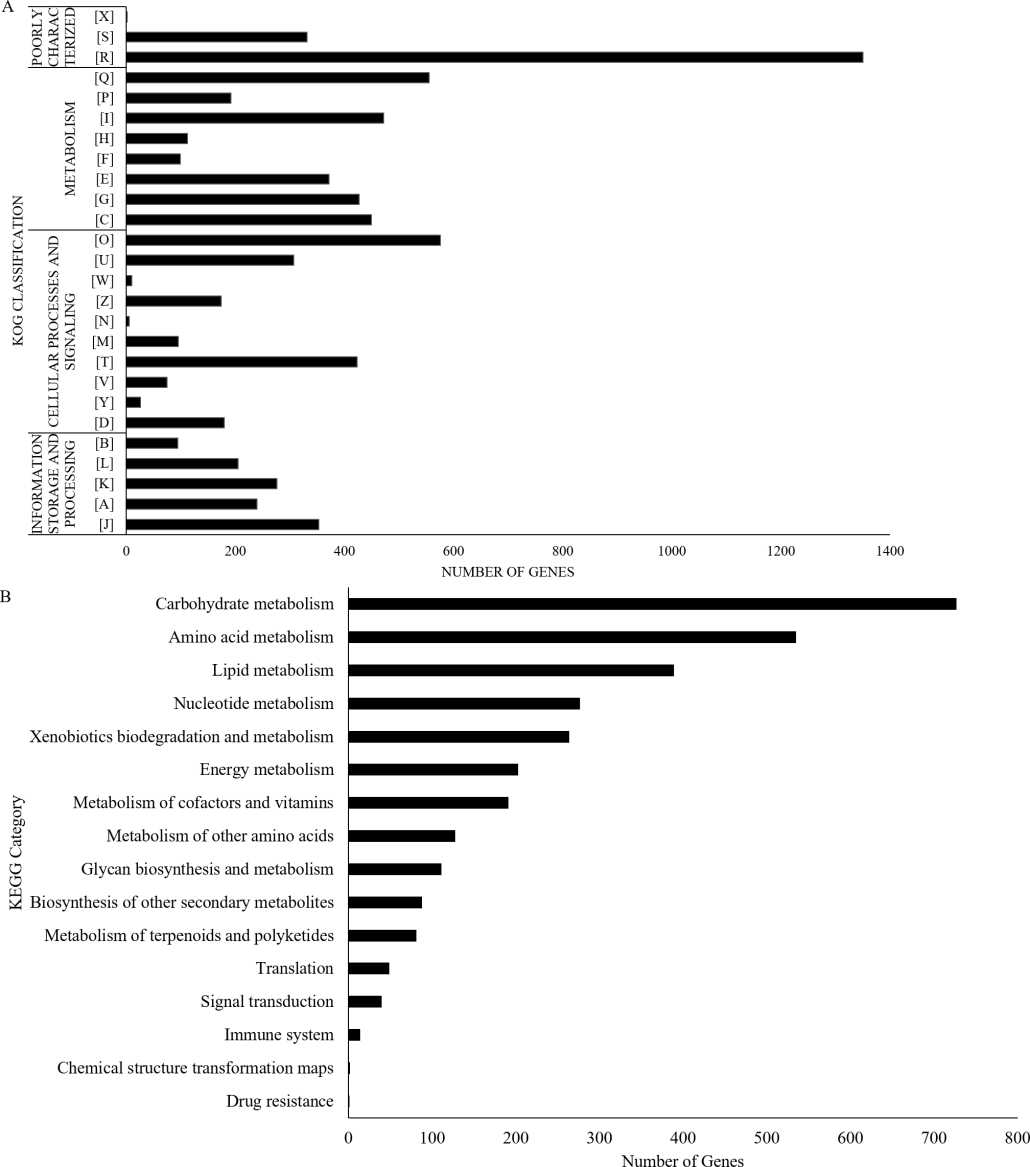
UM 591 gene functions assigned by (A) KOG, and (B) KEGG categories. Legends: [A] RNA processing and modification; [B] Chromatin structure and dynamics; [C] Energy production and conversion; [D] Cell cycle control, cell division, chromosome partitioning; [E] Amino acid transport and metabolism; [F] Nucleotide transport and metabolism; [G] Carbohydrate transport and metabolism; [H] Coenzyme transport and metabolism; [I] Lipid transport and metabolism; [J] Translation, ribosomal structure and biogenesis; [K] Transcription; [L] Replication, recombination and repair; [M] Cell wall/membrane/envelope biogenesis; [N] Cell motility; [O] Posttranslational modification, protein turnover, chaperones; [P] Inorganic ion transport and metabolism; [Q] Secondary metabolites biosynthesis, transport and catabolism; [R] General function prediction only; [S] Function unknown; [T] Signal transduction mechanisms; [U] Intracellular trafficking, secretion, and vesicular transport; [V] Defence mechanisms; [W] Extracellular structures; [X] Unnamed Protein; [Y] Nuclear structure; [Z] Cytoskeleton.

The UM 591 genome contains 13,531 coding genes (>99 bp) with average gene length of 1,420 bp. The gene density in this genome is 3.23 genes per 10 kb sequences with an average of 2.79 exons per gene. A total of 33 rRNAs and 164 tRNAs were predicted in the genome. Protein sequence homology searches in NCBI (nt), Swiss-Prot, and Interpro databases return total of 8,418, 1,692, and 9,824 homologous genes, respectively. Out of the total of 8,418 genes, 6,093 genes encode for hypothetical protein based on the top hit matches in NCBI. Figure 2A summarises the distribution of 7,962 genes in UM 591 according to classes in Gene Ontology (GO).

All the predicted proteins were subjected to KOG database to assign functional classification, which result in 7,399 matches. Top eight classifications with the highest gene counts are: [O] Posttranslational modification, protein turnover, chaperones (576), [Q] Secondary metabolites biosynthesis, transport and catabolism (555); [I] Lipid transport and metabolism (472); [C] Energy production and conversion (449); [G] Carbohydrate transport and metabolism (427); [T] Signal transduction mechanisms (423); [E] Amino acid transport and metabolism (372); and [J] Translation, ribosomal structure and biogenesis (353). These categories are mainly involved in metabolism, cellular processes and signalling, and information storage and processing (Fig. 2B). However, 1,682 genes were poorly characterised, and these genes were distributed under category [R] General function prediction only, [S] Unknown Function, and [X] Unnamed protein (Fig. 2B).

UM 591 genes are subjected to KEGG database to assign molecular pathways, resulting in the assignment of 3,100 genes to known pathways. Top five KEGG pathway with the most abundant gene distributions are carbohydrate metabolism (727), amino acid metabolism (535), lipid metabolism (389), nucleotide metabolism (277), and xenobiotics biodegradation and metabolism (264) (Fig. 2B).

### BUSCO-based quality assessment

To assess the genome assembly and gene set completeness of UM 591, we examined the genome sequence and predicted genes for single-copy orthologs based on BUSCO profiles for fungi. Of the 1,438 genes expected to be present as single copies in a fungal genome, the benchmarking strategy showed that the genome assembly of UM 591 contained a total 1,430 (99.5%) genes, of which 62 (4.6%) were duplicated and 59 (4.4%) were fragmented. Benchmarking on the genes predicted for UM 591 showed that, of 1,438 queried, 1,222 (85.0%) present in single copy, 177 (12.3%) were duplicated while 36 (2.5%) were fragmented. The overall results indicated that the genome assembly and gene prediction of UM 591 are of high degree of completeness (only 8 and 3 BUSCO genes missing in genome and gene set, respectively).

### Virulence genes

Virulence factor is briefly defined as any factors that increases the virulence of a pathogen to invade a host (Hogan, Klein & Levitz, 1996). In this predictive study, we performed homology search using PHI-base database on UM 591 predicted genes to identify genes that could be potential virulence factor. The homology search returns 483 matches to known homologs in the database.

We subjected these homology data to KOG to assign functional classification, resulting in 536 annotated genes, of which 82 genes did not fit any functional classes. The top five KOG classifications are: [X] Unnamed protein (82), [R] General function prediction only (52), [T] Signal transduction mechanisms (48), [I] Lipid transport and metabolism (28), and [O] Posttranslational modification, protein turnover, chaperones (25). Eighty-two percent (438/536) of the annotated genes are assigned into single class of functions while the remaining eighteen percent (98/536) of the genes are assigned into multiple classes of functions.

Virulence factor is one of the critical elements in evaluating pathogenicity. Thus, we screened for potential virulence factors which fulfil the criteria as follows: (A) annotated by PHI-base; and (B) implicated with reduced virulence, loss of pathogenicity, lethal, or hypervirulence status as decribed by PHI-based; and (C) localised extracellularly (secreted protein). We managed to filter out 177 potential virulent factor genes (Table S2), of which 61 of them are homologous to lethal genes (gene silencing causes death of the pathogen) and 10 are hypervirulence gene (gene silencing causes elevated pathogenicity of the pathogen). Among these genes, there are 71 putative genes that may involve in human infection because the experimental host annotated in these data are animal model (mouse or rabbit), although it is also important to note that evidence of infection in animal model does not reflect that these genes will cause infections in human. These genes are homologous to virulence gene of *Aspergillus fumigatus*, *Cryptococcus neoformans*, *Exophiala dermatitidis* and *C. albicans* (Table S2).

**Figure 3:**
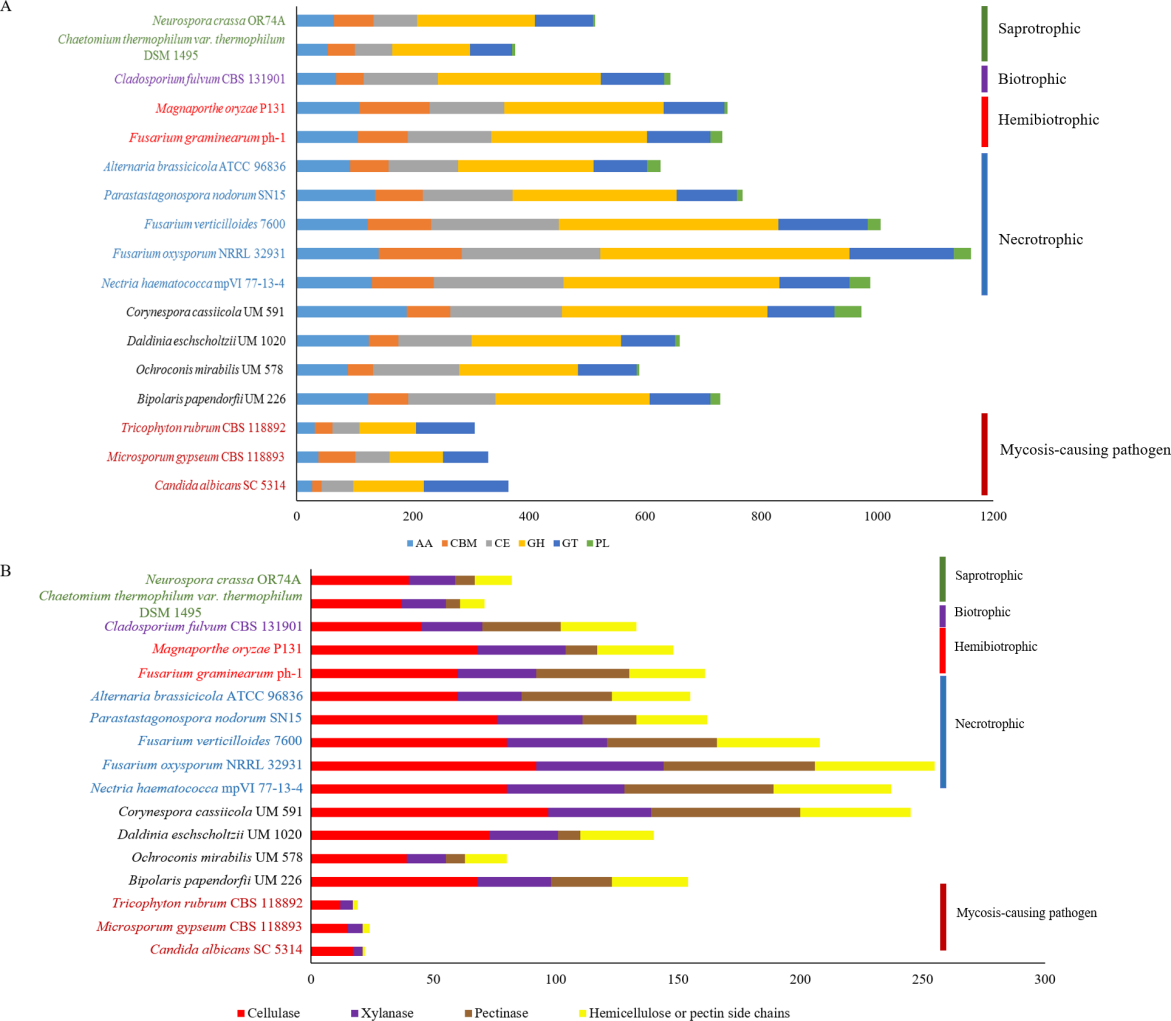
Comparative distribution of CAZymes according to (A) classes of CAZyme module and (B) plant cell wall degrading enzymes. PL, polysaccharide lyases; GT, glycosyl transferases; GH, glycoside hydrolases; CE, carbohydrate esterases; CBM, carbohydrate-binding module and AA, auxiliary activity.

### CAZyme analysis

Plant pathogenesis start with invasion of plants’ cell wall that is made up of complex polysaccharides such as cellulose, hemicellulose, and pectin (Cosgrove, 2005). Plant pathogens produce CAZymes to digest these polysaccharides (O’Connell et al., 2012; Zhao et al., 2014) and in fungi, the enrichment of different types of CAZymes differ in accordance to respective lifestyles (Kohler et al., 2015; Zhao et al., 2014). This variance gives rise to diverse CAZyme profiles; presumably due to natural adaptation to the environment and habitat common to respective fungus. The predicted genes in UM 591 were subjected to CAZyme homology search using dbCAN, resulting in a total of 973 CAZymes predicted. The predicted CAZymes distribution is summarized as follows according to respective classes and gene count: auxiliary activity (AA)—189; carbohydrate esterases (CE)—193, glycoside hydrolases (GH)—354, glycosyl transferases (GT)—115, carbohydrate-binding module (CBM)—75, and polysaccharide lyases (PL)—47. We observed that CE10 (92) CAZymes are the most abundant in UM 591, followed by AA7 (66), CE1 (49), AA3(44), AA9 (41), and GH43 (28). These enzymes are involved in hydrolysis of carbohydrate and non-carbohydrate substrates (CEs and GHs), and oxidative degradation of lignin-based components of plants’ cell wall (AA) (Correia, 2010; Levasseur et al., 2013).

**Figure 4:**
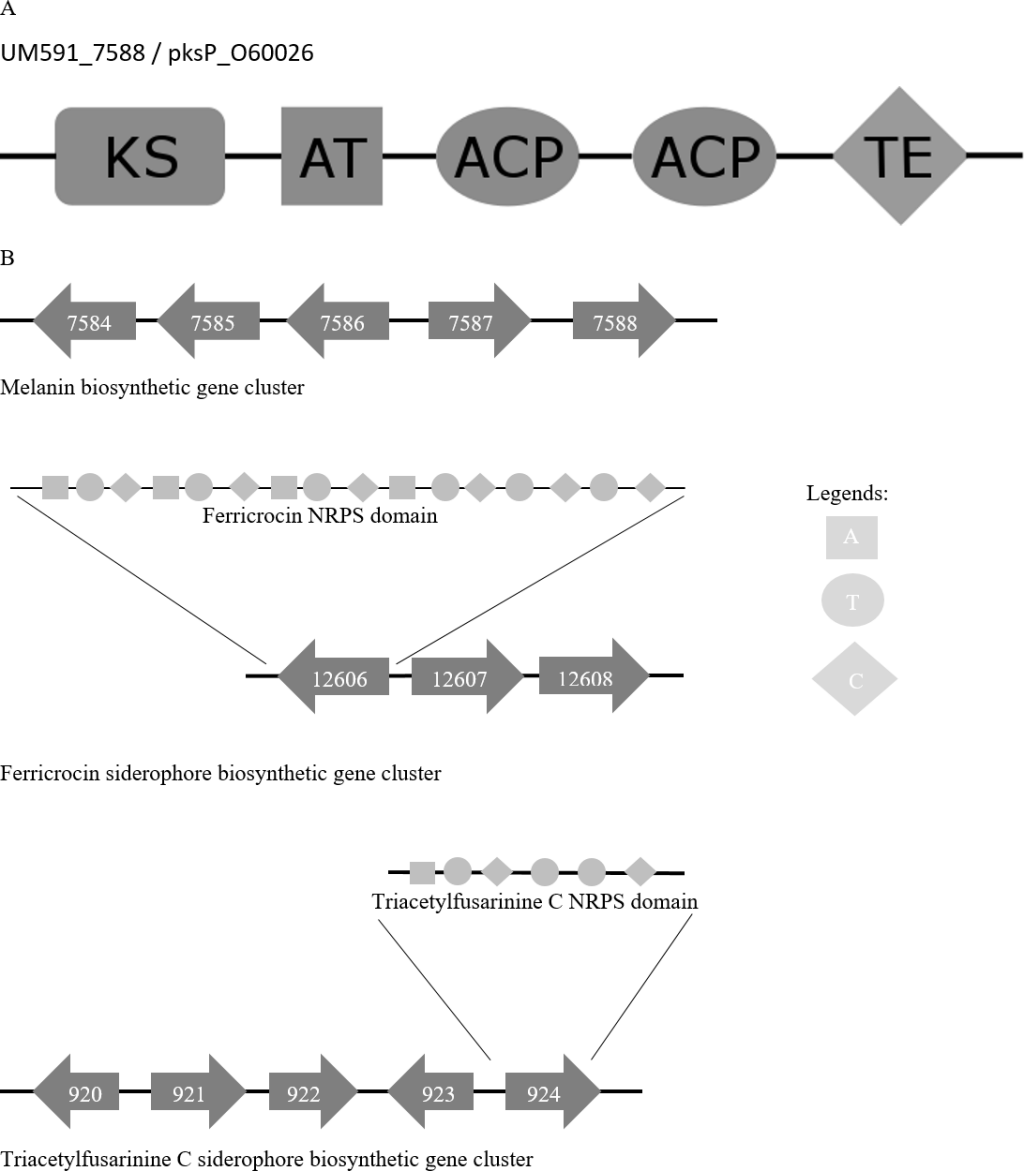
(A) Melanin biosynthesis PKS domain distribution of *A. fumigatus* AF293 (Uniprot accession no.: O60026) and UM591_5788. (B) Representation of putative melanin and siderophore biosynthetis cluster. KS, Ketosynthase; ACP, Acyl carrier protein; AT, Acyltransferase; TE, Thioesterase; A, Adenylation; T, Thiolation; C, Condensation domain.

The overall CAZyme distribution of UM 591 was compared to necrotrophic (*Fusarium oxysporum* NRRL 32931, *Fusarium verticillioides* 7600, *Nectria haematococca* mpVI 77-13-4, *Alternaria brassicicola* ATCC 96836, and *Parastagonospora nodorum* SN15), saprotrophic (*Chaetomium thermophilum var. thermophilum* DSM 1495 and *Neurospora crassa* OR74A), biotrophic (*Cladosporium fulvum* CBS 131901), and hemibiotrophic (*Fusarium graminearum* ph-1 and *Magnaporthe oryzae* P131) fungi to elucidate UM 591 nutritional strategy. CAZyme distribution in UM 591 is comparable to necrotrophic and hemibiotrophic fungi (Fig. 3A). Total pectate lyases, pectinases, and hemicellulose or pectin side chains enzymes (Figs. 3A and 4B) are higher among these fungi compared to saprotrophic and biotrophic fungi. This enrichment is suggestive of UM 591, necrotrophic, and hemibiotrophic fungi in this study possess greater capacity to digest pectin. These fungi also are more enriched in glycoside hydrolases compared to the usual polysaccharide-digestive saprotrophic and biotrophic fungi (Gan et al., 2016; Glass et al., 2013; Ohm et al., 2012). Enrichment in glycoside hydrolases is not surprising in plant pathogens, but the expansion in the modular size of this class of enzyme is suggestive of exceptional plant invasive capability. The most abundant (count of >18 modules) CAZyme module in UM 591 are CE10 (92), AA7 (66), CE1 (49), AA3 (44), AA9 (41), GH3 (25), GH5 (22), GH16 (21), and GT2 (20) (Tables S4 and S5). This CAZyme profile is comparable to three species of necrotrophic fungi (*Fusarium oxysporum*, *Fusarium verticillioides*, and *Fusarium solani* teleomorphic form -*Nectria haematococca*) (Fig. 3A and Table S3). Figure 3B also shows that these four species are more enriched in cell wall degrading enzymes. Our data suggest that UM 591 adopts a necrotrophic lifestyle.

There are 43 genes in UM 591 that are predicted with multiple CAZyme modules per gene (Table S6). These predicted multi-modular CAZymes may suggestive of CAZymes that are capable of more than one enzymatic digestion processes and will require actual experiment to ascertain this finding. Recently, one multi-functional recombinant GH26 enzyme derived from Mehsani buffalo rumen metagenome that is capable of mannase, xylanase, endo-glucanase, and esterase activity has been described (Patel et al., 2016). Although rare, similar multi-functional enzymes also have been reported (Bao et al., 2011; Ko et al., 2011; Zhao et al., 2010).

In plant pathogenesis, three major groups of cell wall degradative enzymes have been extensively studied, namely the lignocellulase, pectinase, and cutinase. In this section, we screened for the putative enzymes by filtering UM 591 CAZymes data according to classes of CAZymes under respective groups of enzymes that were reported in publications. Lignocellulases consist of cellulases (GH1, GH3, GH5, GH45, GH74 and AA9) and xylanases (GH3, GH10, GH11, GH39, GH115 and CE15) which collectively can degrade cellulose, lignin and hemicellulose (Benoit et al., 2015; Morgenstern, Powlowski & Tsang, 2014; Zhao et al., 2014). Based on these classes of CAZymes, we identified 97 putative cellulases and 42 putative xylanases genes (Fig. 3B) in UM 591. A total of 61 putative pectinases genes were identified in UM 591, and these enzymes belong to CE8, GH28, GH78, GH88, PL1, PL3, and PL9 module as described previously (Benoit et al., 2015; Zhao et al., 2014). Recently, Morgenstern, Powlowski & Tsang (2014) introduces three new cellulases from the auxiliary activity class of CAZyme (AA9, AA10, and AA11). These putative copper-dependent lytic polysaccharide monooxygenase (LPMO), but to date only AA9 (formerly GH61) and AA10 (formerly CBM33) have been described in details while AA11 has was recently identified. UM 591 has comparatively high AA9 module (Table S3) than necrotrophic fungi. *C. cassiicola* is well known for causing leaf spot disease on rubber tree (Barhoom & Sharon, 2004) and therefore cutinase is important in leaf invasion. Nine genes of UM 591 are identified to encode cutinases , which belongs to CE5 module (Table S6). The comprehensive distribution of CAZyme modules in all reference fungal species used in this study is available in Table S4 while a summary of some CAZymes of interest is available in  Table S5.

Surface binding is important for effective host invasion and colonisation. In UM 591, six genes (UM591_3966, UM591_4384, UM591_5636, UM591_7223, UM591_12118, and UM591_12120) are homologous to CBM50 modules or also commonly known as Lysin Motif (LysM). LysM is utilised to camouflage fungal cell wall receptor protein, chitin, from detected by plant’s surface chitin receptor and impairs the plant’s immunogenic responses (Kombrink & Thomma, 2013).

Secreted CAZymes are potential virulence factors especially for plants, but to be a valid virulence factor, actual experimentation is required to validate and affirms the designation. In this section, we attempted to screen for potential virulence factors in UM 591’s CAZymes by performing homology search in PHI-base database using the predicted CAZyme genes. In addition, we pay more attention to secreted CAZymes because these enzymes are more likely to be potential virulence factors in a predictive study. Overall, nine and forty-nine CAZymes homologs that were tested on animal and plant models, respectively, were identified. Out of the nine homologs that are tested on animal models, eight genes are homologous to genes of *A. fumigatus*, *Exophiala dermatitidis*, and *C. albicans* that are implicated with reduced virulence or lethal status in gene silencing experiments. Only UM591_8532 is a putative secreted GH72 CAZyme and it is homologous to *A. fumigatus* GEL2 virulence gene. On the other hand, only 19 homologs that were tested on plant models are implicated with reduced virulence, loss of pathogenicity, and hypervirulence status in gene silencing experiments. These homologs originate from *F*. *oxysporum*, *M. oryzae*, *Mycosphaerella graminicola*, *Monilinia fructicola*, *Nectria haematococca*, *Colletotrichum gloeosporioides*, *Colletotrichum coccodes*, *Trichoderma virens*, and *Cladosporium fulvum*. From these nineteen genes, eleven are secreted CAZymes, of which four are predicted with high-cysteine content. The three genes that are homologous to the hypervirulence genes of *Monilinia fructicola* are UM591_2724, UM591_6311, and UM591_7102. These gene encodes for CE5 cutinases which have been discussed in CAZyme section previously. A comprehensive description of virulence-related CAZymes is available in Table S2.

### Secreted peptidase and lipases

Secreted peptidases play important roles in signalling, nutrition procurement, and degradation of host tissue (Ohm et al., 2012). Putative secreted peptidases are identified in two steps. First, UM 591 genes are screened for secreted peptides using SignalIP and subsequently, the secreted peptide genes are subjected to homology search using MEROPS (Table S7). A total of 216 peptidases are annotated in UM 591, of which 69 are secreted peptidases. There are two major groups of peptidases identified in UM 591, namely metallopeptidases (84); which consist of M10 (A), M12 (A), M14 (A), M28 (A and B), M35, M36, and M43 (B) family, and serine peptidase (66); which consist of S1 (A and E), S8 (A), S9 (B and C), S10, S12, S33, and S53 family peptidases. One of the group of secreted peptidases that are responsible for skin pathogenesis are keratinases (Monod et al., 2002; Reichard et al., 2006), and known keratinases are identified in UM 591. These keratinases are described in accordance to MEROPS class designation and peptidase homologs as follows: S8 (subtilisin), M35 (deuterolysin, penicillolysin), M36 (fungalysin), S1 (trypsin), S9 (DPPIV and DPPV) and S33 (Tripeptidyl-peptidase B). Subtilisin family peptidases (S8) have been reported capable of disrupting the integrity of keratinaceous substrate (Huang et al., 2015), aid in conidial attachment of *Microsporum canis* onto animal and human (Băguţ et al., 2012; Baldo et al., 2008). The types of keratinases produced by fungi varies depending on the types of keratinaceous surface the fungi adhere (Chen et al., 2010; Huang et al., 2015). Subtilisin (SUB1 to SUB7), leucine aminopeptidase (LAP1 and LAP2), and dipeptidyl peptidase (DPPIV and DPPV) are few of the peptidases that are selectively produced (Chen et al., 2010; Huang et al., 2015). In UM 591, UM591_4361 is homologous to LAP1 (81% identical, Swiss-Prot) while UM591_11691 is homologous to LAP2 (54% identical, Swiss-Prot). Both homologs had been annotated by MEROPS as Mername-AA063 peptidase of *A. fumigatus* and *Pseudomonas*-type secreted aminopeptidase of *Pseudomonas aeruginosa*, respectively. Carboxypeptidases (M14A, S10) and aminopeptidases (M28A) are commonly studied in human pathogenic fungi as well and these peptidases are also identified in UM 591 (Monod et al., 2002; Sriranganadane, 2011) (Table S7). Myroilysin (UM591_9269) which digest elastin is annotated. This peptidase belongs to M10A family and it is homologous to myroilysin of deep sea bacterium *Myroides profundi* (Yang et al., 2015). Interestingly, UM591_9269 also matched to nematode astacin 35 peptidase (UM591_9269) of M12A family. This peptidase has been proposed to digest cuticular collagen (Park et al., 2010). Sulfite efflux pump encoded by SSU1 gene had been proposed to aid keratin digestion by transporting sulfite to keratin for cleavage of cysteine-disulfide bridges prior to keratinase digestion (Chinnapun, 2015). In UM 591, we identified two SSU1 genes (UM591_737 and UM591_10590).

Other than peptidases, two classes of peptidase inhibitors are annotated by MEROPS, which are peptidase A inhibitor 1 of I09 family (UM591_11255 and 7170) and serine carboxypeptidase Y inhibitor of I51 (UM591_2665 and 3685) family. Peptidase A inhibitor 1 inhibits subtilisin family peptidases while serine carboxypeptidase Y inhibitor inhibits some of serine endopeptidase of S1 family. Interestingly, peptidase encoded by UM591_11255 was predicted to have a subtilisin domain, which may harbour proteolytic as well as inhibiting activities. A comprehensive description of peptidases is available in Table S7.

In order to identify putative lipases, UM 591 genes are subjected to homology search using Lipase Engineering database, returning with a total of 105 genes which are homologous to six types of lipases, three types of hydrolases, four types of esterases, cutinase, dipeptidyl peptidase IV-like, lysophospholipase and lysosomal protective peptide-like enzymes (Table 2).

**Table 2:** Summary of lipases in UM 591 retrieved from homology search using Lipase Engineering database. A comprehensive description of lipases is available in Table S8.

Type of enzyme	Enzyme description	Number of genes
Cutinase	Cutinases	4
Dipeptidyl peptidase-like	Dipeptidyl peptidase IV like	3
Esterase	Bacterial esterases abH13	3
	Carboxylesterases	7
	Thioesterases	1
	Xylanase esterases	10
Hydrolase	Cytosolic Hydrolases	5
	Dienlactone Hydrolases	2
	Microsomal Hydrolases	12
Lipase	Burkholderia lipases	1
	Candida rugosa lipase-like	18
	Filamentous fungi lipases	1
	Gastric lipases	3
	Lysophospholipase	1
	Moraxella lipase 2 like	25
	Moraxella lipase 3 like	2
Lysosomal protective protein like	Lysosomal protective protein like	7
Total number of genes		105

Peptidases and lipases are important enzymes for dermatophytes to invade skin layer, and both are potential virulence factors. Therefore, we analysed both peptidases and lipases data alongside PHI-base, NCBI (nt), Swiss-Prot and Interpro data. Two potential virulence peptidases are identified (Table S7). UM591_6187 is homologous to *SPM1* of *Magnaporthe oryzae* (64% identical, PHI: 2117). SPM1 is a cytosolic serine protease that plays an important role in conidiation and autophagy in fungal infection (Chen et al., 2010). UM591_8185 is homologous to *Ss-ggt1* of *Sclerotinia sclerotiorum* (55% identical, PHI: 2411). Ss-ggt1 govern the development of sclerotia and appressorium by recycling glutathione to maintain the balance of redox environment (Li, Liang & Rollins, 2012). Research on fungal lipases have proven that lipases are species-specific virulence factors (Bender & Flieger, 2010), such as FGL1 of *Fusarium graminearium* (Voigt, Schäfer & Salomon, 2005), LipA of *Burkholderia glumae* (Devescovi et al., 2007) and the proposed virulent NhL1 of *Nectria haematococca* mating population VI (Eddine, Hannemann & Schafer, 2001). In this section, we predict potential virulence factor in lipases based on pathogen-host interaction description annotated by PHI-base and criteria (secreted protein and PHI-base virulence status) previously described in virulence gene section. Five secreted lipases are identified in UM 591 (Table S8). Three cutinase genes (UM591_2724, UM591_6311, and UM591_7102) are homologous to *MfCUT1* hypervirulence genes (52%, 50% and 52% identical, respectively; PHI: 2383) of *Monilinia fructicola*. A hypothetical secreted Moraxella lipase 3-like gene (UM591_6979) are homologous to *TMPL* of *Alternaria brassicola* (55% identical, PHI: 2296). This gene is transmembrane protein that regulate redox homeostasis and is an important component for development of conidia in *A. brassicicola* (Kim et al., 2009). A putative secreted GH10 family xylanase esterase (UM591_7944) is homologous to endo-1, 4-beta-xylanase gene of *M. oryzae* (63% identical, PHI: 2204).

### Secondary metabolites

In fungi, genes involved in biosynthesis of a particular secondary metabolite are usually located close to one another, forming a series of genes which as a whole, contribute to production of a secondary metabolite product and hence considered as a cluster of genes (Chen et al., 2007; Wight, Labuda & Walton, 2013; Yu et al., 2004). Besides that, there are selected few known secondary metabolite biosynthesis genes that does not operate in one cluster of genes, but are separated into multiple cluster or series of sequential genes in the genome but these clusters are involved in production of a specific secondary metabolite product (Eliahu et al., 2007; Wawrzyn, Bloch & Schmidt-Dannert, 2012). In this study, 50 backbone genes encoding possible secondary metabolite synthetases or synthases are identified using the SMURF tool for analysis. A total of 38 secondary metabolite gene clusters were predicted based on the predicted PKS and NRPS backbone genes. Potential cluster of genes is screened manually with reference to the predicted backbone genes via descriptive searching with annotations from NCBI, Swiss-Prot, and Interpro databases. In addition to that, secondary metabolites genes which are descriptively annotated but not predicted as one of the gene clusters are investigated as well. Here, we identified putative melanin, siderophore, terpene (*ent*-kaurene and lycopene), and toxins (sterigmatocystin, cercosporin, HC-toxin, and gliotoxin) biosynthesis genes and clusters as summarized in  Table S9.

#### Melanin biosynthesis

Melanin has been reported to protect fungi from UV, oxidative, and chemical stresses (Jacobson, 2000). Melanin is an important virulence factor that aid in host penetration by the generation of turgor pressure to exert pressure on plant cell wall for penetrative purpose (Howard & Ferrari, 1989). With reference to *Cochliobolus heterostrophus* PKS18 (Eliahu et al., 2007) (NCBI accession no.: AAR90272) and three critical genes involved in melanin biosynthesis that was described in *Alternaria alternata* (Kimura & Tsuge, 1993), we managed to identify a putative melanin biosynthesis gene cluster in UM 591 (Table S9). The cluster (UM591_5784 to UM591_5788) consist of two unknown genes (UM591_7586 and UM591_7587) and two critical components (melanin PKS and tetrahydroxynaphthalene reductase) out of the usual six-gene cluster which has been well studied in other fungi (Eliahu et al., 2007; Pihet et al., 2009; Woo et al., 2010). The important third component, scytalone dehydratase, is predicted (UM591_76 and UM591_10618). Gene distribution in the cluster are comparatively similar to that of *Magnaporthe grisea* (Eliahu et al., 2007) and PKS domain motif is the same as Pksp/Alb1 of *A. fumigatus* (Fig. 4). Thus, melanin is postulated to be biosynthesized using DHN-melanin biosynthesis pathway in UM 591.

#### Siderophore biosynthesis

Two putative siderophore clusters: intracellular ferricrocin and extracellular triacetylfusarinine C are identified in UM 591 with reference to *C. heterostrophus* NPS2 and NPS4 (Turgeon & Bushley, 2009; Schrettl et al., 2007). The putative intracellular ferricrocin cluster (UM591_12606 to UM591_12609) is made up of NPS2/sidC homolog NRPS (UM591_12606), sidA ornithine monooxygenase (UM591_12608), and sidL acyl-CoA N-acyltransferase (UM591_920 or UM591_11221) (Blatzer et al., 2011; Schrettl et al., 2007) (Table S9). Probable gene encoding sidL was identified by searching for genes in UM 591 that were annotated by Interpro with the same set of domains from known sidL (UniProt accession no: Q4WJX7) (Blatzer et al., 2011). The predicted amino acid sequences of UM591_12606 are subjected to InterproScan to identify the NRPS modular motif of this core gene. The motif generated is ATC-ATC-ATC-ATC-TC-TC, which is consistent with the domain architecture observed in other Dothideomycetes’ NPS2 (Haas, Eisendle & Turgeon, 2008). The second siderophore cluster (UM591_920 to UM591_924) that encodes putative extracellular triacetylfusarinine C synthase consist of NPS6/sidD homolog (UM591_924), sidG GNAT family acetyltransferase (UM591_923), sidF acyl-CoA N-acyltransferase (UM591_920), and sidA ornithine monooxygenase (UM591_921). The amino acids sequences of UM591_924 and sidD (UniProt accession no: Q4WF53) (Schrettl et al., 2007) are subjected to InterproScan to elucidate the NRPS motif, and the result shows same domain pattern and distribution in both proteins.

#### Terpene biosynthesis

Sesquiterpenes are commonly produced by fungi and its gene distributions in the biosynthesis cluster may vary from having one core synthases to multiple synthases or having multiple cytochrome P450 genes (Wawrzyn, Bloch & Schmidt-Dannert, 2012). The backbone gene is identified by Interpro domain search for terpene cyclase conserved domain, and the gene cluster was identified by searching for presence of nearby P450 gene and neighbourhood genes that could be made up of transporter, regulatory, other synthase, and other biosynthetic genes (Wawrzyn, Bloch & Schmidt-Dannert, 2012). UM 591 genome encodes a great span of genes with P450 domain (223 genes) distanced from one to another by up to 300 genes. This allows great flexibility of non-clustered terpene/terpenoid synthesis if relevant synthase is present in the vicinity.

We identified a putative *ent*-kaurene and a lycopene biosynthesis cluster by screening for terpene cyclase and synthase domains. Putative *ent*-kaurene cluster (UM591_10047 to UM591_10055) consists of two synthase genes (UM591_10052 and UM591_10054) (Table S9). Both genes could possibly encode for either copalyl diphosphate synthase (CPS) or *ent*-kaur-16-ene synthase (KS). Copalyl diphosphate is an intermediate component for biosynthesis of *ent*-kaurene which, in turn, is an intermediate component of gibberellin biosynthesis (Hedden & Kamiya, 1997). Gibberellin biosynthesis pathway requires additional gene of gibberellic acid (GA_4_) desaturase (*des*) and four P450 classes of oxidases (GA_14_ synthase (P450-1), GA_20_ oxidase (P450-2), and C_13_-oxidase (P450-3)) (Rim et al., 2013) aside from genes found in an *ent*-kaurene gene cluster. *des*, P450-1, P450-2, and P450-3 genes are not specifically annotated in UM 591 genome data, but there is one P450 gene (UM591_10049) within the putative *ent*-kaurene cluster. As discussed previously, UM 591 have a great span of P450 gene across the whole genome. Hence, the function of the other three P450 components (P450-1, P450-2, and P450-3) may be easily substituted. P450-4 and geranylgeranyl pyrophosphate (GGPP) synthase were identified about 3,000 genes downstream from the cluster (UM591_13501 and UM591_10561, respectively), which suggestive that this putative gibberellin/*ent*-kaurene biosynthesis may involve more than one cluster of genes. Interestingly, there is one transcription factor at each end of the cluster, suggesting that gene transcription may initiate from either direction. Based on the arrangement of genes in the putative *ent*-kaurene cluster (Lengeler et al., 2000), transcription initiated from the regulator at downstream is more likely whereby copalyl diphosphate is first produced from geranyl pyrophosphate catalysed by CPS followed by production of *ent*-kaurene catalysed by KS and subsequently, oxidation by P450 oxidases.

Lycopene biosynthesis cluster (UM591_12052 to UM591_12055) consist primarily of phytoene dehydrogenase (*car*B), bi-functional phytoene synthase (*car*RA) (Arrach et al., 2001); which perform both functions of lycopene cyclase and phytoene synthesis; and two set of carotenoid oxygenases (Table S9). In UM 591, only one carotenoid oxygenase (*car*X) is found within the cluster (UM591_12053, 55% identical, UniProt accession no: Q5GN50). *Car*X was proposed to catalyse similar aldehyde dehydrogenation as *Neurospora crassa*’s ylo-1 enzyme to produce neurosporaxanthin (Avalos, Prado-Cabrero & Estrada, 2012; Jin, Lee & Lee, 2010). A light-sensing rhodopsin (*car*O) (Thewes et al., 2005) is identified within the cluster but *car*T which is usually found in the carotenoid biosynthesis cluster (Jin, Lee & Lee, 2010; Prado-Cabrero et al., 2007) could be encoded by UM591_3196 (44% identical, Uniprot accession no: A1KQY4). This gene is identified based on amino acid identity and carotenoid domain (IPR004294) as annotated by Interpro.

#### Toxin biosynthesis

We predicted a great range of toxin biosynthesis pathway in the UM591 genome (Table S9). Aflatoxin is commonly affiliated to food contamination (Hammami et al., 2014) and has been extensively studied in *Aspergillus* spp. (Yu et al., 2004). Aflatoxin and sterigmatocystin biosynthesis pathways may share majority of the biosynthesis protein since sterigmatocystin is the precursor to produce aflatoxin (Yu et al., 2004). We managed to identify majority of the aflatoxin biosynthesis pathway components including omtB/ aflO/ stcP, which produces sterigmatocystin, and ordA/ aflQ, which produces the final product aflatoxins (Yu et al., 2004). We also identified putative omtA/aflP in UM 591 by amino acid sequence identity and domain search with reference to omtA (UniProt accession no: P55790) of *Aspergillus flavus*, showing that UM 591 may be able to synthesize aflatoxin aside from  sterigmatocystin.

A putative cercosporin cluster (UM591_7760 to UM591_7765) is identified, but the two usual cercosporin biosynthesis pathway components, CTB6 and CTB7, could not be found within this cluster of genes (Chen et al., 2007). The closest matching gene for CTB7, a FAD/FMN-dependent oxidoreductase, is UM591_1286 with reference to the known domain of CTB7 (UniProt accession no: A0ST45). CTB6 encodes for putative NADPH-dependent oxidoreductase, but there are no specific matches based on protein nomenclature, similar keywords search (such as CTB6, Cercospora, and cercosporin), or by domain matching because this protein is commonly conserved in living organisms.

The majority of core gliotoxin biosynthesis pathway components are identified in UM 591 except for P450 oxidoreductase gliF, glutathione S-transferase gliG, dipeptidase gliJ, and o-methyltransferase gliM (Gardiner & Howlett, 2005). Alternatively, we screened for possible candidate genes for these missing components by domain search with reference to gli gene cluster data from *A. fumigatus* (Cramer et al., 2006; Gardiner & Howlett, 2005) and from *Aspergillus* and Aspergillosis Website (Atherton, 2014). Other than transcription factor, gliotoxin biosynthesis also is regulated by laeA and gipA (Dolan et al., 2015). UM591_507 (80% identical) encodes for a laeA-like peptide of *C. heterostrophus*, but gipA is not found. gliZ, gliC, gliA, gliN, and gliP are identified in a cluster (UM591_12893, UM591_12898, UM591_12902, UM591_12903, and UM591_12905, respectively; Table S9) as predicted by SMURF. The missing gliF could be functionally substituted either by a probable monooxygenase (UM591_12904, 49% identical, NCBI nt accession no: GAA90272) in the cluster or by wide ranges of P450 oxidoreductases/monooxygenases identified in UM 591.

HC-toxin was well described particularly in *Cochliobolus* and *Alternaria* species (Wight, Labuda & Walton, 2013). Here, we predicted three probable genes encoding for HC-toxin synthetases (UM591_6434, UM591_11373, and UM591_13673). The majority of HC-toxin biosynthesis components are identified by domain search with reference to TOXD, TOXF, and TOXG (Uniprot accession no: P54006, Q9Y885, and Q9UW18, respectively). However, no match for TOXC is found, and the only fatty acid synthase beta subunit gene in UM 591 is UM591_668, which is one of the predicted components in aflatoxin biosynthesis. However, the domain annotated by Interpro for this gene is missing the starter unit ACP transacylase (IPR032088) domain that is found in TOXC/AjTOXC (Uniprot accession no: Q92215 and S5FIF0), but two additional domains, PKS acyl transferase (IPR020801) and aldolase-type TIM barrel (IPR013785), are found in UM591_668, suggesting that this protein may serve more than one functions.

## Conclusion

In this study, we characterises *C. cassiicola* UM 591 from genomic perspective to understand the underlying factors that aid in its pathogenicity. CAZymes profiling has shown that *C. cassiicola* adopts necrotrophic lifestyle. This finding agrees with the necrotic aftermath observed on leaves, soft fruit, and even human skin infected by *C. cassiicola*. In addition, the similarity of CAZyme profile between UM 591, *F. oxysporum, F. solani*, and *F. verticillioides* reflects that the gene expression and the infectivity approach of these four fungi maybe similar. *Fusarium* spp. are well known pathogens in both human and plant, and it is not surprising to observe CAZyme enrichment in plant pathogens. Despite so, the expanded capacity in CAZymes compared to saprotrophic and biotrophic fungi denotes that UM 591 can be highly invasive in plants, since the top three CAZyme classes (CE, AA, and GH) collectively can degrade various polysaccaharides and non-carbohydrate substrates. The predicted cutinases that are homologous to hypervirulence gene signify that cutinases in UM 591 may be an important virulence factor in plant. Many known keratinases are predicted in UM 591, and this finding denotes that mycoses due to UM 591 may not be merely opportunistic events, or rather it may be as pathogenic as any dermatophytes. Nevertheless, many factors could still dampen the chances of infection, for example the proposed thermal barrier due to human physiological temperature, personal and workplace hygiene, and wound management. It is also important to note that although *C. cassiicola* is an opportunistic fungus, this fungus do causes serious necrotic lesion and the infection could expand to wide area of the skin. We managed to narrow down to quite a number of potential virulence factors in CAZymes and secreted peptidases with annotations from PHI-base and MEROPS databases. The predicted secondary metabolites are also good evidences that reflects the invasive potential and stress-tolerance nature of the fungus. Melanin, siderophores, and terpenes can protects the fungus from destructive stresses while toxins provide opportunities for invasion and colonization.

##  Supplemental Information

10.7717/peerj.2841/supp-1 Supplemental Information 1 List of fungal species used in the study

10.7717/peerj.2841/supp-2 Supplemental Information 2 Virulence-related gene with reference to CAZyme, MEROPS and Lipase Engineering data

10.7717/peerj.2841/supp-3 Supplemental Information 3 CAZyme profile based on the most abundant modules in UM 591

10.7717/peerj.2841/supp-4 Supplemental Information 4 Comprehensive CAZyme module comparison among fungi used in the study

10.7717/peerj.2841/supp-5 Supplemental Information 5 Summary of CAZyme gene of interest in this study All data were based on ¿50% identity as annotated by NCBI nr and Swiss-Prot database.

10.7717/peerj.2841/supp-6 Supplemental Information 6 Summary of CAZyme virulence factor gene with description from MEROPS, Lipase Engineering database and Phi-base

10.7717/peerj.2841/supp-7 Supplemental Information 7 Peptidases annotation with reference to PHI-base, NCBI nr, Swiss-Prot and Interpro data

10.7717/peerj.2841/supp-8 Supplemental Information 8 Peptidases annotation with reference to PHI-base, NCBI nr, Swiss-Prot and Interpro data

10.7717/peerj.2841/supp-9 Supplemental Information 9 Predicted putative secondary metabolite gene clusters

## Additional Information and Declarations

### DNA Deposition

The following information was supplied regarding the deposition of DNA sequences: Whole Genome Shotgun project at NCBI/Genbank under the accession JAQF00000000.1.

### Data Availability

The following information was supplied regarding data availability: The raw data has been supplied as a Supplementary File.
